# Retrocaval Ureter Diagnosed Following Point-of-Care Ultrasound Detection of Hydronephrosis: A Case Report

**DOI:** 10.5811/cpcem.66161

**Published:** 2026-10-26

**Authors:** Noah Marzook, Mostafa Alhabboubi, Ahmad Koshak, Abdullah Basnawi, Yasser Ajabnoor, Suha Albadr

**Affiliations:** * University of Jeddah, Faculty of Medicine, Department of Pediatrics, Jeddah, Saudi Arabia; † University of Jeddah, Faculty of Medicine, Department of Family Medicine, Jeddah, Saudi Arabia; ‡ University of Tabuk, Faculty of Medicine, Department of Internal Medicine, Division of Emergency Medicine, Tabuk, Saudi Arabia; § King Abdullah University of Science and Technology, International Medical Clinic, Department of Radiology, Thuwal, Saudi Arabia

**Keywords:** retrocaval ureter, hydronephrosis, point-of-care ultrasound, abdominal pain, case report

## Abstract

**Introduction:**

Retrocaval ureter (RCU) is a rare congenital anomaly resulting from abnormal development of the inferior vena cava, leading to ureteral obstruction and hydronephrosis. Patients often present with nonspecific symptoms, making diagnosis challenging. Point-of-care ultrasound (POCUS) is frequently used in the emergency department (ED) to evaluate abdominal pain and may identify clinically important abnormalities requiring further investigation.

**Case Report:**

A 30-year-old woman presented to the ED with a two-day history of nonspecific abdominal pain. Physical examination was notable only for mild epigastric tenderness. Abdominal POCUS performed by the emergency physician demonstrated moderate right-sided hydronephrosis. The patient had no urinary symptoms and normal renal function. Follow-up renal ultrasound confirmed hydronephrosis and proximal hydroureter without nephrolithiasis. Contrast-enhanced computed tomography subsequently revealed a RCU with the characteristic fish-hook configuration. Renography demonstrated preserved renal function with delayed drainage secondary to ureteral obstruction. The patient underwent surgical correction with ureteral reconstruction and stent placement.

**Conclusion:**

This case highlights the value of POCUS in the evaluation of patients with nonspecific abdominal pain. Detection of hydronephrosis prompted further diagnostic evaluation and led to the diagnosis of a rare congenital urologic anomaly requiring surgical intervention. Emergency physicians should consider further investigation when hydronephrosis is identified on POCUS, even in the absence of urinary symptoms.

## INTRODUCTION

Abdominal pain is one of the most frequent complaints of patients presenting to the emergency department (ED), accounting for 5–10% of ED visits.^1^ Abdominal point-of-care ultrasound (POCUS) enhances diagnostic accuracy and streamlines the clinical workup for patients presentingwith abdominal pain.^2^ In this case report, we describe a patient presenting to the ED with nonspecific abdominal pain, where abdominal POCUS was done as a part of the emergency physician’s routine assessment. This led to the eventual diagnosis of a retrocaval ureter (RCU), a rare congenital urogenital abnormality for which surgical intervention is required. To the best of our knowledge, no previous cases of RCU have been reported in the literature in the context of using POCUS.

## CASE REPORT

A 30-year-old woman presented to the ED with a two-day history of abdominal pain. She had no fever, vomiting, or diarrhea. On physical examination, her vitals were normal; the only notable finding was mild epigastric tenderness. Abdominal POCUS revealed right renal pelvis dilation. The patient did not report any urinary symptoms. Laboratory results showed a normal urinalysis; creatinine was 66 μmol/L (reference range, 45–90 μmol/L), and the calculated glomerular filtration rate was 110 mL/min/1.73 m^2^ (90–120 mL/min/1.73 m^2^). The physician considered the finding of hydronephrosis as potentially incidental and discharged the patient with a preliminary diagnosis of gastritis. A follow-up outpatient renal ultrasound was arranged to evaluate the hydronephrosis further and determine its underlying cause.

Two weeks later, the renal ultrasound showed moderate right-sided hydronephrosis with proximal hydroureter and no detectable renal stones (Image 1). The subsequent week, the patient was seen by a urologist, to whom she continued to complain of persistent abdominal pain. A contrast computed tomography (CT) (Image 2) showed persistent moderate hydronephrosis and proximal third hydroureter, with the renal pelvis reaching 2.2 cm in diameter. The proximal ureter deviated medially and posteriorly relative to the inferior vena cava (IVC), descending anterior to the aortocaval groove, displaying a positive “ fish-hook sign.” The patient was diagnosed with RCU.

Further investigation with a renogram showed preserved right kidney but delayed drainage due to ureteral compression of the right kidney. The patient underwent a right ureteral transposition, resection, and ureteroureterostomy over an indwelling double-J stent to correct the abnormality.

*CPC-EM Capsule*

What do we already know about this clinical entity?

*Retrocaval ureter is a rare congenital anomaly in which the ureter passes behind the inferior vena cava, causing obstruction and hydronephrosis.*

What makes this presentation of disease reportable?

*The anomaly was found incidentally when point-of-care ultrasound (POCUS) showed hydronephrosis in a patient with nonspecific abdominal pain and no urinary symptoms.*

What is the major learning point?

*Hydronephrosis on POCUS warrants further imaging even without urinary symptoms; it can reveal surgically correctable anomalies such as retrocaval ureter.*

How might this improve emergency medicine practice?

*Using abdominal POCUS in undifferentiated abdominal pain can prompt earlier imaging, diagnosis, and timely referral for treatable urologic anomalies.*

## DISCUSSION

Retrocaval ureter is a rare congenital urogenital anomaly. The ureters typically descend laterally to the IVC. In patients with RCU, the ureter descends in a spiral path around the IVC. The proximal ureter passes posterior to the IVC at the L3 level to lie medially, while the distal ureter returns to its normal course, crossing from medial to lateral anterior to the lower IVC. It results from the abnormal persistence of the right subcardinal vein ventral to the ureter during IVC embryogenesis.^3^ Even though it is a congenital defect, patients usually present in the third or fourth decade of life with a history of abdominal pain due to ureteric obstruction and associated hydronephrosis.^4^ Prolonged ureteric obstruction can lead to obstructive uropathy, affecting renal function.^5^ The clinical presentation of RCU is nonspecific, and its diagnosis relies primarily on radiological imaging, particularly contrast-enhanced CT.^6^

Hydronephrosis is often the first finding to prompt further investigation, but it is nonspecific and may lead to diagnostic uncertainty or misinterpretation.^4^ Several imaging modalities can raise suspicion of an RCU. The hallmark feature commonly associated with RCU diagnosis is the fish-hook sign, also known as the reversed J-shaped ureter, which demonstrates a characteristic curvature of the proximal right ureter as it courses behind the IVC. This sign can be seen on intravenous pyelogram, retrograde pyelography, or CT urography.^5^ The inability to confirm or exclude RCU on CT scan frequently results from inadequate opacification of the distal ureter. This limitation is usually due to obstruction or urinary stasis, which prevents clear visualization of the ureteral course, particularly in its distal segment. In such cases, placing a ureteral stent can improve diagnostic accuracy by opacifying the entire ureter, clarifying its course, and reducing unnecessary surgical interventions.^7^

In our case, the right proximal ureter is seen on CT coursing medial and posterior to the IVC at the L3–L4 (retrocaval course) level, with narrowing of the ureter as it passed behind the IVC. On ultrasound, RCU may be suggested when a transverse scan demonstrates a dilated ureter coursing posterior to the IVC. The use of color Doppler is valuable in this context because it helps differentiate the proximal ureter from adjacent vascular structures, specifically the right renal vein and the IVC.^6^,^8^ Surgery is required in patients with persistent symptoms or worsening renal function. Repair usually includes the resection of the redundant RCU segment, interposition, and anastomosis.^7^

Abdominal POCUS can be used to identify various intraabdominal pathologies in patients presenting with abdominal pain, including hydronephrosis, as seen in our case.^8^,^9^ The use of POCUS to identify hydronephrosis is one of the more commonly used applications. A meta-analysis of five studies (*N* = 1,773) comparing CT to hydronephrosis detected on POCUS showed a pooled sensitivity and specificity of 70.2% and 75.4%, respectively, with a calculated positive and negative likelihood ratio of 2.85 and 0.39.^10^ While a negative test does not rule out the possible presence of hydronephrosis, a positive POCUS may aid in decision-making and further care of patients.

## CONCLUSION

The early detection of hydronephrosis on POCUS triggered further imaging that identified a rare congenital anomaly, leading to timely surgical intervention. The use of POCUS in identifying hydronephrosis is reliable. While a negative result does not exclude hydronephrosis, a positive finding may support clinical decision-making and guide further patient care. This case report identifies a rare entity that should be in the differential diagnosis for undifferentiated abdominal pain and for unexplained hydronephrosis.

## Figures and Tables

**Image 1:**
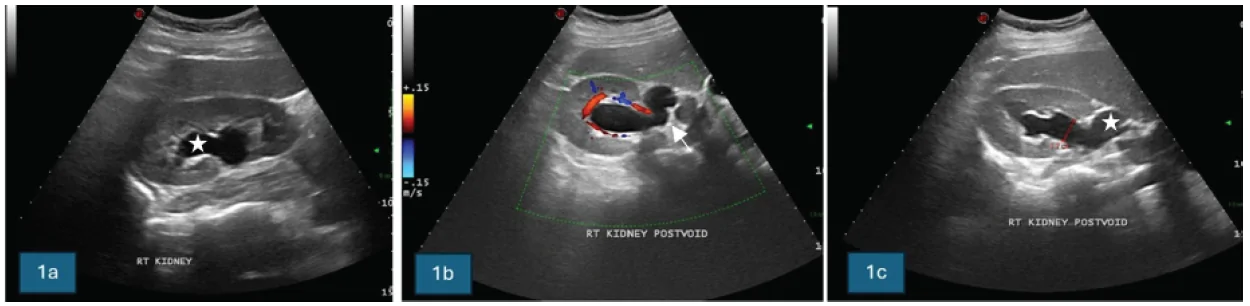
Long-axis (**A**) and short-axis (**B,C**) ultrasound images of the right kidney demonstrating moderate hydronephrosis and proximal hydroureter (star). Image **B** reveals acute angulation at the ureteropelvic junction (arrow).

**Image 2:**
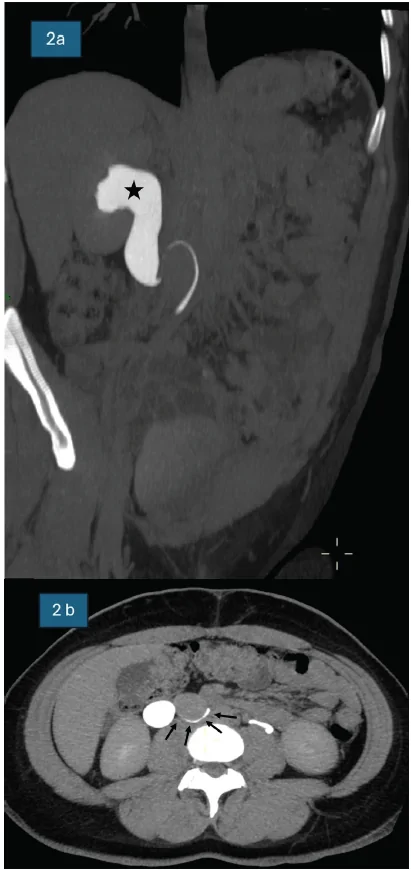
Delayed contrast-enhanced computed tomography urogram with oblique multiplanar reformations. (**A**) Coronal image demonstrating moderate right hydronephrosis and proximal hydroureter (star) secondary to obstruction, with the proximal ureter following a retrocaval course medial and posterior to the inferior vena cava (IVC). (**B**) Axial image at the third and fourth lumbar level showing the right proximal ureter coursing posterior to the IVC, with focal narrowing behind the vein (arrows) consistent with a retrocaval ureter.
